# Porphyrin-Based Compounds: Synthesis and Application

**DOI:** 10.3390/molecules28207108

**Published:** 2023-10-16

**Authors:** Carlos J. P. Monteiro, M. Amparo F. Faustino, Carlos Serpa

**Affiliations:** 1 LAQV-Requimte and Department of Chemistry, University of Aveiro, 3810-193 Aveiro, Portugal; 2 CQC-IMS, Department of Chemistry, University of Coimbra, Rua Larga, 3004-535 Coimbra, Portugal

Porphyrin-based compounds are an attractive and versatile class of molecules that have attracted significant attention across different scientific disciplines [1]. These unique molecules, characterized by their distinctive macrocyclic structure featuring four pyrrole-type rings linked by methine bridges, have drawn the attention of chemists, biologists, and material scientists [2]. Porphyrins are not only notorious for their crucial roles in biological systems, serving as the core of heme in hemoglobin and chlorophyll in photosynthesis [3], but also for their remarkable capacity to undergo synthetic modification [4,5], leading to a wide range of functionalized derivatives. The synthesis and modification potential of porphyrins, combined with their extraordinary electronic and photophysical properties, has paved the way for an extensive possibility of applications, spanning from biomedical applications [6,7,8], to catalysis [9], sensors [10,11], energy conversion [12,13], and advanced materials [14]. The current Special Issue, entitled “Porphyrin-Based Compounds: Synthesis and Application”, features 15 original research papers and one comprehensive review. These contributions are all dedicated to exploring the synthesis and functionalization of tetrapyrrolic macrocycles and their applications across several fields.

The importance of panchromatic absorbers and their potential applications motivated Bocian, Holten, Lindsey and co-workers [15] to develop an efficient synthetic procedure to obtain two panchromatic triads with absorption ranging from 350 to 700 nm and fluorescence emission from 733 to 743 nm. These triads comprise two perylene-monoimides connected to a porphyrin molecule through an ethyne unit. Additionally, these triads also contain a single anchoring group, either an alkynoic acid or an isophthalate unit for surface attachment, offering versatility in their use. The key steps in the synthesis involve the preparation of *trans*-AB-porphyrins, which are then coupled with the perylene-monoamide groups through copper-free Sonogashira-coupling reactions. The authors highlighted that these triads demonstrate significantly broader absorption spectra and enhanced fluorescence when compared to their individual components.

Paolesse and co-workers [16] reported a synthetic strategy to access zinc (II) phthalocyanine containing a 2-(2,4-dichloro-benzyl)-4-(1,1,3,3-tetramethyl-butyl)phenoxy group. They initiated this process from the Clofoctol derivative, a synthetic compound with antiviral activity against SARS-CoV-2. The synthesis involved the preparation of phthalonitrile precursors through nucleophilic aromatic nitro displacement of 2-(2,4-dichloro-benzyl)-4-(1,1,3,3-tetramethylbutyl)phenol (Clofoctol) with 4-nitrophthalonitrile. Subsequently, cyclotetramerization took place in the presence of zinc(II) salts to form the corresponding zinc(II) phthalocyanine complex, which was shown to be soluble in several organic solvents. Furthermore, the paper also explored the photochemical and electrochemical properties of the new compound and assessment as a solid-state sensing material in gravimetric chemical sensors, making it a promising material for gas sensing.

Moyano and co-workers [17] presented an efficient method for the synthesis of amino-functionalized porphyrins. These amino compounds were initially investigated as a new class of bifunctional catalysts for asymmetric organophotocatalysis. Two variants of amine-porphyrin hybrids were generated: one involving a cyclic secondary amine connected to a β-pyrrolic position (referred to as Type A) and another linked to the *p*-position of a *meso* phenyl ring (referred to as Type B), along with their corresponding Cu(II) metalated derivatives. These synthetic steps involved the condensation or reductive amination reactions with appropriate chiral amines. Furthermore, an additional Type B bifunctional catalyst, 5,10,15-triphenyl-20-((*S*)-4-((pyrrolidine-2-carboxamido)methyl)phenyl)porphyrin, was achieved via the amidation of 5-(4-(aminomethyl)phenyl)-10,15,20-triphenylporphyrin with *N*-Boc-*L*-proline. The authors evaluated the potential use of Type A amine-porphyrin hybrids as asymmetric, bifunctional organophotocatalysts in a Diels–Alder cycloaddition reaction.

Rebelo and co-workers [18] contributed to the sustainable functionalization of renewable aromatic compounds. They reported one-pot oxidation reactions at room temperature, employing environmentally friendly H_2_O_2_ as an oxidant and ethanol as a solvent, along with an electron-withdrawing iron (III) porphyrin catalyst, used in a low catalyst loading (<2 mol%). The mechanistic aspects of these transformations were also investigated, offering insights into the reaction pathways. The investigation primarily centered on three inherently stable aromatic compounds: acridine, *o*-xylene, and quinoline. The results revealed an unconventional initial epoxidation of the aromatic ring catalyzed by this system. The study evidenced a distinctive preference for *o*-xylene oxidation, as it occurred exclusively on the aromatic ring and not on the methyl groups.

The synthesis and evaluation of porphyrin photosensitizers (PS) as antimicrobials have also been assessed by different authors in this Special Issue. In this context, Uliana and co-workers [19] elucidated the semisynthesis of PS derived from chlorophyll *a*, with several primary aliphatic amines (butylamine, hexylamine, and octylamine), to produce different derivatives and their utilization in antimicrobial photodynamic therapy (aPDT) toward different microorganisms such as *Staphylococcus aureus*, *Escherichia coli*, and *Candida albicans*. The modifications introduced in the porphyrin aimed to improve the solubility and amphiphilicity of the PSs to enhance their performance in aPDT. The purpurin-18 derivatives containing carboxylic acid groups were particularly effective against *S. aureus* and *C. albicans*, achieving significant inactivation. The length of the carbon chain in the PS influenced their performance, as longer chains led to reduced PS uptake by the microorganism and subsequently lower photoinactivation outcomes.

Monteiro, Faustino, and co-workers [20] published findings on the synthesis and antimicrobial action of a novel class of porphyrin-based PS incorporating sulfonamide groups and a zinc(II) complex. These compounds were evaluated to assess their effectiveness in aPDT against methicillin-resistant *Staphylococcus aureus* (MRSA) when combined with potassium iodide (KI) as a co-adjuvant. All examined compounds demonstrated the capacity to generate ^1^O_2_, with the zinc(II) complex exhibiting the highest efficiency. The research also assessed the formation of iodine (I_2_) in the presence of potassium iodide (KI) and each PS under white light irradiation. The results highlighted the effective generation of I_2_ by all compounds, with the sulfonic acid derivative (TPP(SO_3_H)_4_) demonstrating the most notable efficiency. The photodynamic action of these PS was evaluated against MRSA. All compounds displayed significant photoinactivation of MRSA, with TPP(SO_3_H)_4_ emerging as the most effective. The zinc(II) complex, ZnTPP(SO_2_NHEt)_4_, also exhibited promising antimicrobial activity. These findings highlight the considerable potential of these PS, especially when used in combination with KI, in aPDT, addressing the pressing challenge of antimicrobial resistance.

Boscensu, Burloiu, Socoteanu and co-workers [21], investigated the effects exerted in vitro of three asymmetrical porphyrins, (5-(2-hydroxyphenyl)-10,15,20-tris(4-acetoxy-3-methoxyphenyl)porphyrin, 5-(2-hydroxyphenyl)-10,15,20-tris(4-acetoxy-3-methoxyphenyl)porphyrinatozinc(II), and 5-(2-hydroxyphenyl)-10,15,20-tris(4-acetoxy-3-methoxyphenyl)porphyrinatocopper(II)), on human U937 cell membranes. The porphyrins were studied for their impact on transmembrane potential and membrane anisotropy using fluorescent probes. The findings indicate that porphyrins induce cell membrane hyperpolarization and increase membrane anisotropy, suggesting enhanced rigidity. This could alter membrane protein interaction, impacting cellular function. Molecular docking simulations suggest that these derivatives may interact with membrane proteins such as SERCA2b, Slo1, and KATP channels, possibly modulating their activity. The research suggests that these asymmetrical porphyrins have notable effects on cell membranes and may hold promise for further study as potential agents for different biomedical applications. Further experimental validation is required to confirm these interactions and their impact on cellular homeostasis.

Chaves, Serpa and co-worker [22] explored the interaction between human serum albumin (HSA) and the non-charged synthetic porphyrin, 5,10,15,20-tetra(pyridin-4-yl)porphyrin (4-TPyP), evaluated via in vitro assays under physiological conditions using spectroscopic techniques (UV-vis, circular dichroism, steady-state, time-resolved, synchronous, and 3D-fluorescence) combined with in silico calculations via molecular docking. The UV-vis and steady-state fluorescence parameters indicated a ground-state association between HSA and 4-TPyP and the absence of any dynamic fluorescence quenching was confirmed by the same average fluorescence lifetime for HSA without and with 4-TPyP. Therefore, the Stern–Volmer quenching (K_SV_) constant reflects the binding affinity, indicating a moderate interaction being spontaneous, enthalpically, and entropically driven. Binding produces only a very weak perturbation on the secondary structure of albumin. There is just one main binding site in HSA for 4-TPyP (n ≈ 1.0), probably in the subdomain IIA (site I), where the Trp-214 residue can be found. The microenvironment around this fluorophore seems not to be perturbed even with 4-TPyP interacting via hydrogen bonding and van der Waals forces with the amino acid residues in the subdomain IIA. To offer a molecular-level explanation of the binding HSA: 4-TPyP, molecular docking calculations were also carried out for the three main binding sites of albumin (subdomains IIA, IIIA, and IB).

BODIPY (boron-dipyrromethene) represents a class of synthetic dyes or fluorophores recognized for their tunable properties, such as absorption and emission wavelengths, achieved through chemical and structural modifications. In this context, Vicente and co-workers [23] undertook a study focused on synthesizing and characterizing a range of 8(*meso*)-pyridyl-BODIPY compounds featuring diverse substituents. The study encompassed the synthesis of BODIPY compounds incorporating 2-, 3-, or 4-pyridyl groups, along with functionalization involving nitro and chlorine groups at the 2,6-positions. Additionally, analogs with 2,6-methoxycarbonyl groups were synthesized. The study systematically investigated the structure and spectroscopic properties of these compounds using experimental and computational methods. Introducing 2,6-methoxycarbonyl groups significantly boosted fluorescence quantum yields of BODIPYs in polar organic solvents. Conversely, adding a single nitro group reduced fluorescence and caused shifts in absorption and emission bands to shorter wavelengths. Interestingly, incorporating a chloro substituent partially restored fluorescence and induced shifts to longer wavelengths. This research provides insights into how electron-withdrawing groups affect the photophysical properties of 8(*meso*)-pyridyl-BODIPYs, offering valuable information for potential applications.

Iglesias and co-workers [24] investigated the photophysical and photobiological properties of a series of *trans*-C_6_F_5_ *meso*-substituted corroles. These corroles have different groups attached to their *meso* positions (phenyl, naphthyl, 4-(hydroxy)phenyl, or 4-(thiomethyl)phenyl)) and are of interest for potential applications in PDT and other photo-related processes. The study involved characterizing these compounds using various techniques such as electrochemical methods and photophysical properties, including absorption and emission characteristics, as well as their behavior in different solvents. Theoretical calculations were performed to gain insights into their properties. The research suggests that these substituted corrole compounds show promise for various photoinduced processes and prefer binding to DNA in the minor grooves, as well as interacting with HSA.

The work reported by Ruhilman, Choua, Ruppert and co-workers [25] comprehensively investigated the synthesis and electronic properties of nickel(II) porphyrins with bulky nitrogen donors at the *meso* positions, prepared using Ullmann methodology or Buchwald–Hartwig amination reactions to establish new *C-N* bonds. They also conducted electrochemical studies, spectroelectrochemical measurements, electron paramagnetic resonance (EPR) studies, and density functional theory (DFT) calculations to gain insights into the electronic properties and delocalization of radical cations in these compounds. Electron paramagnetic resonance (EPR) and electron nuclear double resonance spectroscopy (ENDOR) were used to study the extent of delocalization of the generated radical cations. The results indicated that the radical cation distribution varied depending on the specific compound and the nature of the substituents. The study also explored the magnetic properties of some compounds, including the formation of biradicals and their exchange interactions. Additionally, DFT calculations supported the EPR spectroscopic data.

Peverati and Morgante [26] analyzed the performance of 250 electronic structure theory methods (including 240 density functional approximations) for the description of spin states and the binding properties of iron, manganese, and cobalt porphyrins. The assessment employed the Por21 database of high-level computational data. The results showed that the approximations failed to achieve the “chemical accuracy” target of 1.0 kcal/mol by a long margin. The best-performing methods achieved a mean unsigned error (MUE) < 15.0 kcal/mol, but the errors were at least twice as large for most methods. Semilocal functionals and global hybrid functionals with a low percentage of exact exchange were found to be the least problematic for spin states and binding energies, in agreement with the general knowledge in transition metal computational chemistry. These results reflect both an intrinsic difficulty of density functional calculations on metalloporphyrin and the difficulties in obtaining reliable reference energies and experimental results.

Golleti and co-workers [27] highlighted the importance of chirality in organic materials, using Reflectance Anisotropy Spectroscopy (RAS) to investigate porphyrins and porphyrin-related compounds, in various experimental conditions, including ultra-high vacuum-controlled atmospheres and liquid. The study introduced a technical enhancement to the RAS spectrometer called CD-RAS (circular dichroism RAS), which allows for the measurement of circular dichroism (CD) in samples under right- and left-circularly polarized light in transmission mode, offering flexibility and potential applications in various experimental setups. The research emphasizes the significance of chirality in organic materials and the role of porphyrins in exploring this phenomenon.

Exploring novel compounds and synthetic pathways for medical applications led Pęgier and co-workers [28] to optimize the synthesis of copper complexes with various water-soluble porphyrins (5,10,15,20-tetrakis(1-methylpyridinium-4-yl)porphyrin tetratosylate (TMPyP), 5,10,15,20-tetrakis(4-sulfonatophenyl)porphyrin (TSPP), and also 5,10,15,20-tetrakis(4-carboxyphenyl)porphyrin (TCPP)) for potential use in radiopharmaceuticals, with a particular emphasis on applications in positron emission tomography (PET). Efforts were focused on developing the fastest possible method that would meet the requirements of labeling with short-lived copper isotopes used in PET. Two optimized methods were applied for the synthesis of the ^64^Cu–porphyrin complex: (i) using ascorbic acid (AA) as a reducing agent for accelerated room temperature complexation; and (ii) utilizing microwave-assisted synthesis at 140 °C for 1–2 min. Both methods are efficient for application in radiopharmaceutical synthesis as they are one-step, fast, and avoid the use of toxic or harsh chemicals requiring separation.

Moscoso, Pedrosa and co-workers [29] introduced an innovative colorimetric and fluorescent probe designed for the detection of glutathione (GSH) within a pH 7.4 phosphate buffer solution. This probe is built upon a highly luminescent porphyrin analog, specifically a carboxylated pyrrolidine-fused chlorin (TCPC). The probe runs on the principle of forming a TCPC- Hg^2+^ complex, where TCPC binds to mercury ions (Hg^2+^), resulting in a notable reduction in its fluorescence output. However, when GSH is introduced into the system, it competes with Hg^2+^ for binding to TCPC, leading to an increase in fluorescence intensity. This fluorescence enhancement serves as the basis for GSH detection. The sensitivity of this detection method depends on the concentration ratio of TCPC to Hg^2+^, with a remarkable achievement of a detection limit as low as 40 nM under specific ratios. The study further highlights the selectivity of the sensor, as it demonstrates the ability to discriminate against potential interferents, including various metal ions and cysteine.

In this Special Issue, an important contribution is a comprehensive review authored by Boscescu, Socoteanu, Burloi, Ferreira and co-workers [30] focusing on porphyrin macrocycles and their potential in cancer diagnosis and therapy. The paper shed light on photodynamic therapy (PDT) as a non-invasive therapy with minimal side effects compared to conventional cancer treatments. The properties of porphyrin macrocycles, including their absorption and emission spectra, were discussed in detail. The authors also presented a critical overview of the main commercial PS, followed by short descriptions of some strategies approached in the development of third-generation PS. The paper discussed challenges in solubility and molecular aggregation for tetrapyrrolic compounds, emphasizing efforts to enhance their clinical performance through modifications and functionalization. Strategies for functionalizing porphyrinic macrocycles with bioactive molecule fragments such as amino acids, peptides, and sugars to optimize the biodistribution of PS were also comprehensively explored and discussed.

To sum up, the Special Issue entitled “Porphyrin-Based Compounds: Synthesis and Application” provides a current perspective on the synthesis and modification of porphyrins, metalloporphyrins, and related compounds. Considering the challenges in this exciting field, this edition not only enhances our understanding of synthetic methodologies but also unveils a wide range of impactful applications. These applications cover critical domains such as biomedical applications (including radiopharmaceuticals, DNA and protein interactions, cancer therapeutics, and microbial inactivation) as well as catalysis, sensors, advanced materials, and computational studies. The editors wish to thank the invited authors for their engaging and insightful contributions. They anticipate that the joint insights presented in this Special Issue will have a significant impact on the field, enriching research, development, and knowledge concerning the applications of porphyrins and their analogs.
